# Draft Genome Sequences of Pathotype Strains for Three Pathovars Belonging to Three Xanthomonas Species

**DOI:** 10.1128/MRA.00923-18

**Published:** 2018-09-27

**Authors:** Vassiliki A. Michalopoulou, Joana G. Vicente, David J. Studholme, Panagiotis F. Sarris

**Affiliations:** a Institute of Molecular Biology and Biotechnology, Foundation for Research and Technology–Hellas, Heraklion, Crete, Greece; b School of Life Sciences, Warwick Crop Centre, University of Warwick, Warwick, Wellesbourne, United Kingdom; c Biosciences, College of Life and Environmental Sciences, University of Exeter, Exeter, United Kingdom; Iowa State University

## Abstract

We present here the draft genome sequences of type/pathotype strains for three Xanthomonas species and pathovars with different host specificities, the Hedera helix L. pathogen Xanthomonas hortorum pv. hederae WHRI 7744 (NCPPB 939T), the rice pathogen X. oryzae pv. oryzicola WHRI 5234 (NCPPB 1585), and the cotton pathogen X. citri subsp. malvacearum WHRI 5232 (NCPPB 633).

## Abstract (precis)

We present here the draft genome sequences of type/pathotype strains for three Xanthomonas species and pathovars with different host specificities, the Hedera helix L. pathogen Xanthomonas hortorum pv.

## ANNOUNCEMENT

The genus Xanthomonas comprises a diverse and economically important group of bacterial pathogens, some of which are quarantine pathogens that cause bacterial spots, rots, wilts, blights, and cankers of plants, leaves, stems, and fruits on a wide variety of plant species (1, 2). The majority of the pathogenic Xanthomonas species reveal high degrees of host specificity, and some are divided into multiple pathovars based on their host specificity. The formal description of each species includes a type strain, and each pathovar has a designated pathotype strain.

Xanthomonas hortorum pv. hederae causes bacterial leaf spot and stem canker on English ivy (Hedera helix L.), as well as on plants of the genera Dizygotheca, Schefflera, Brassaia, Polyscias, and Fatsia and on the species Fatshedera araliaceous. It was originally described in France in 1920 (3, 4). Vauterin et al. in 1990 (5) proposed the reclassification of this pathovar from X. campestris to X. hortorum pv. hederae based on DNA hybridization, metabolic similarities, and protein profiles. The type strain of the species X. hortorum and pathotype strain of X. hortorum pv. hederae (WHRI 7744 = NCPPB 939 = ICMP 453) originates from the United States in 1943.

Bacterial leaf streak (BLS) caused by X. oryzae pv. oryzicola was first reported in the Philippines in 1918 and is present in tropical and subtropical Asia, including China, Malaysia, India, and Indonesia, and also in northern Australia and West Africa (6–8). Several studies, based on DNA fingerprinting, revealed high variability among X. oryzae pv. oryzicola strains (7, 8). The pathotype strain (WHRI 5234 = NCPPB 1585 = ICMP 5743) is from Malaysia from 1964.

X. citri subsp. malvacearum causes bacterial blight of cotton plants (9). This is one of the most devastating bacterial diseases that plague cotton plants worldwide. X. citri subsp. malvacearum has a wide range of aggressiveness depending on the host species (10, 11), and 19 physiological races have been identified. Race 1 is widespread in Australia, India, and the United States, and race 18 is present in Australia, Nicaragua, and India. Races 6, 7, and 10 were reported in Nigeria, and races 1, 2, 8, 21, 26, and 32 were reported in Syria (9). The pathotype strain (WHRI 5232 = NCPPB 633 = ICMP 5739) is from Sudan and was obtained in 1958.

The Xanthomonas strains were routinely grown in nutrient agar broth (peptone A, 6.0 g/liter; beef extract, 1.0 g/liter; yeast extract, 2.0 g/liter; sodium chloride, 5.0 g/liter; pH, ∼7.3) with aeration (180 rpm) at 28°C. The genomic DNA was extracted according to bacterial genomic DNA isolation using cetyltrimethylammonium bromide (12). The genomes of the three above-mentioned strains were sequenced using the Illumina MiSeq next-generation sequencing platform in the University of Exeter Sequence Centre (http://sequencing.exeter.ac.uk) to generate 2.6 million, 1.5 million, and 1.6 million pairs of 300-nucleotide reads, respectively, for X. hortorum pv. hederae, X. oryzae pv. oryzicola, and X. citri subsp. malvacearum.

The sequences were trimmed with TrimGalore (https://github.com/FelixKrueger/TrimGalore), which is a wrapper around CutAdapt version 1.15 (https://cutadapt.readthedocs.io/en/latest), and assembled using the SPAdes version 3.11.1 (13). Total lengths of the three assemblies were 5.52 Mb, 5.24 Mb, and 4.68 Mb. The *N*_50_ contig lengths were 41.6 kb, 81.4 kb, and 61.1 kb. The numbers of contigs were 444, 283, and 179. G+C contents of each genome assembly were 64, 63, and 64%. The assembled sequences were annotated by the NCBI Prokaryotic Genome Annotation Pipeline (14).

### Data availability.

These genome assemblies and the raw sequence reads have been deposited in DDBJ/EMBL/GenBank and in the Sequence Read Archive under the accession numbers PYJG00000000 and SRR7643104, respectively, for X. hortorum pv. hederae WHRI 7744; PYJI00000000 and SRR7643312, respectively, for X. oryzae pv. oryzicola WHRI 5234; and PYJH00000000 and SRR7642345, respectively, for X. citri subsp. malvacearum WHRI 5232.
